# Sleep duration and prostate cancer-specific mortality in the Southern Community Cohort Study

**DOI:** 10.1007/s10552-026-02256-8

**Published:** 2026-10-21

**Authors:** Danica C. Anukam, Roch A. Nianogo, Onyebuchi A. Arah, Jianyu Rao, Jay H. Fowke, Mia Hashibe, Zuo-Feng Zhang

**Affiliations:** 1 Department of Epidemiology, UCLA Fielding School of Public Health, Los Angeles, CA USA; 2 Department of Statistics and Data Science, UCLA College of Letters and Science, Los Angeles, CA USA; 3 Department of Public Health, Aarhus University, Aarhus, Denmark; 4 Department of Pathology and Laboratory Medicine, UCLA David Geffen School of Medicine, Los Angeles, CA USA; 5 Department of Preventive Medicine, The University of Tennessee Health Science Center, College of Medicine, Memphis, TN USA; 6 Jonsson Comprehensive Cancer Center, UCLA, Los Angeles, CA USA

**Keywords:** Sleep duration, Racial disparities, Prostate cancer, Southern Community Cohort Study (SCCS), Prospective cohort

## Abstract

**Purpose:**

Sleep patterns may affect metabolic and behavioral processes, which subsequently could be linked to cancer mortality. However, the relationship between sleep patterns and prostate cancer-specific mortality remains unclear. We evaluated the association of sleep and prostate cancer-specific mortality and investigated whether race/ethnicity influences the association.

**Methods:**

The Southern Community Cohort Study (SCCS), a prospective cohort study, recruited participants from 12 Southeastern states between March 2002 and September 2009. The cohort included 34,166 males, predominantly Black/African American. Sleep exposures were measured via a baseline questionnaire. Cox Proportional hazards models were used to estimate associations between sleep and prostate cancer-specific mortality and were adjusted for important potential confounders.

**Results:**

During follow-up (median 15.9 years), 225 men died from prostate cancer. Longer sleep duration (≥ 9 hours) on weekends versus 7–8 hours was suggestively associated with an increased risk of prostate cancer-specific mortality (hazard ratio (HR)= 1.44, 95% confidence interval (CI): 1.02–2.03). When stratified by race, longer sleep duration was suggestively associated with an increased risk of prostate cancer mortality for both Black and White men, although the associations were insignificant. Overall sleep duration and restless sleep were not associated with prostate cancer-specific mortality.

**Conclusion:**

Men reporting ≥ 9 hours of weekend sleep had an elevated risk of prostate cancer-specific mortality compared with those sleeping 7–8 hours. Stratified analysis suggests an increased risk of prostate cancer-specific mortality among both Black men and White men with ≥ 9 hours of sleep versus 7–8 hours, though estimates were imprecise due to small event numbers.

**Supplementary Information:**

The online version contains supplementary material available at https://doi.org/10.1007/s10552-026-02256-8.

## Introduction

Sleep is vital for one’s health and daily functioning, and insufficient sleep may be associated with increased risk of non-communicable diseases such as obesity, depression, diabetes, and even cancer [1]. With the increasing burdens of cancer and poor sleep, any links between sleep and cancer become even more critical. Prostate cancer is the most common cancer among men and the second leading cause of cancer deaths among men in the United States [2, 3]. The projected new deaths from prostate cancer in the United States in 2026 are 36,320 [2]. While survival rates for prostate cancer patients are relatively higher than for other cancers, it is still a serious disease because of its high incidence and mortality.

Some of the current evidence on sleep and prostate cancer incidence found no consistent association. A meta-analysis conducted by Liu et al*.* found that sleep duration was not associated with prostate cancer risk [4]. However, in our most recent published study, we observed that compared with optimal sleep duration (7–8 hours), shorter sleep duration (< 6 hours) was suggestively associated with a decreased risk of prostate cancer in the overall cohort and among Black men when stratified by race [5]. Findings from studies on sleep medications and poor sleep quality based on actigraphy found associations with greater prostate cancer risk [6, 7].

The role of sleep duration and prostate cancer mortality is not well understood. Very few epidemiological studies have been conducted to investigate the relationship between sleep and prostate cancer-specific mortality, and most studies found no consistent association [7–11]. For instance, Dickerman et al*.* found no clear association between sleep duration, sleep quality, and shift work with prostate cancer mortality [10]. Tan et al. found no association between sleep disturbances (insomnia symptoms and use of hypnotics) and prostate cancer mortality [11]. However, one study, Gapstur et al*.*, did find an elevated risk of fatal prostate cancer during the first 8 years of follow-up among short sleepers ≤ 6 h [12].

In addition to the sparse findings, there is minimal literature on sleep and prostate cancer-specific mortality among Black men. In earlier times, race/ethnicity was not considered in sleep research, but over time, there has been an emerging awareness that race/ethnicity plays a pivotal role in sleep studies [13]. Previous research has suggested that racial and ethnic minorities are disproportionately affected by sleep and circadian disparities [14]. In one study, Ruiter et al*.* conducted a meta-analysis to investigate normal sleep in African Americans and Caucasian Americans and found that in the United States, African American adults reported shorter sleep duration across the 14 studies analyzed in comparison to White adults [15]. In addition, African Americans obtained approximately 28 minutes of less sleep as observed on polysomnography compared to Whites [15]. Regarding prostate cancer, African American/Black men suffer disproportionately from this disease. In fact, African American/Black men have a 2.1 times higher mortality rate compared with non-Hispanic White men [2], and these disparities are greatest in low-income communities. 

Therefore, to address the gap in literature, our study aimed to estimate the magnitude of the association between sleep duration, restless sleep, and prostate cancer-specific mortality in the Southern Community Cohort Study (SCCS), which is a predominantly low-income and racially diverse prospective cohort. Another aim was to evaluate racial disparities in the association of sleep duration and restless sleep with prostate cancer mortality, stratified by Black and White men.

## Methods

### Study design & study population

SCCS is a population-based prospective cohort study, and the methods have been described in detail previously [16, 17]. In brief, study participants were recruited from 12 Southeastern states (Alabama, Arkansas, Florida, Georgia, Kentucky, Louisiana, Mississippi, North Carolina, South Carolina, Tennessee, Virginia, and West Virginia). Recruitment began in March 2002 through September 2009. The study participants were predominantly African American/Black men (67%), and nearly 35,000 male participants were enrolled. About 85,000 men and women ages 40–79 years were eligible for inclusion if they were not diagnosed or under treatment for cancer within the past year (exception of non-melanoma skin cancer), English-speaking, and could provide informed consent. Approximately 85% of participants were recruited from community health centers, and 15% were recruited through population-based methods. Participants who were recruited from community health centers were interviewed at baseline using a computer-assisted interview by a trained interviewer. The remaining participants were recruited by mail from the general population. At baseline, questions included demographic characteristics, lifestyle factors, and socioeconomic status. All study participants provided written consent at enrollment, and this study was approved by the Institutional Review Boards of Vanderbilt University and Meharry Medical College.

For the current study, the analysis was restricted to males, and we examined all males at baseline with the following exclusion criteria: men reporting having prostate cancer prior to baseline (*n*=598). After exclusion, our analytic sample was 33,568 men with 22,437 Non-Hispanic Black/African American men, 9,609 Non-Hispanic White/Caucasian men, and 1,215 men of other racial groups.

### Exposure: sleep assessment

Sleep duration is the primary exposure variable. Sleep duration was asked in a questionnaire at baseline and at follow-up 1 (2008–2013), but in our study, we focused on baseline only. At baseline, SCCS evaluated sleep duration by asking participants about total sleep in a 24-h period. The question was: “How many hours do you typically sleep in a 24-h period?” This question was asked for weekdays and weekends separately, using full-hour increments. The weighted average sleep duration of weekdays and weekends per 24 h was then calculated as follows: [(weekday sleep duration × 5) + (weekend sleep duration × 2)/7], in which sleep duration was indexed to the closest hour and rounded to a decimal place. Sleep duration (weekday, weekend, and weighted average) was classified into four categories: <6 h/day (short sleep), 6 h/day, 7–8 h/day, and ≥ 9 h (long sleep), and 7–8 h/day was used as the reference group. Participant responses who did not know or who refused to answer the question were recoded as the ‘Missing/Unknown’ data category for that specific variable.

Restless sleep is the other exposure variable of interest. Participants were asked at baseline how they were feeling during the past week: “my sleep was restless” with the following options: ‘rarely or none of the time,’ ‘some of the time,’ ‘much of the time,’ and ‘most or all of the time.’ ‘Rarely or none of the time’ was the reference group. Participants with “Refuse” or “Don’t Know” responses were recoded as missing.

### Outcome: prostate cancer-specific mortality

The study outcome was prostate cancer-specific mortality, which was determined via linkage to the National Death Index (NDI) through 31 December 2022. Prostate cancer deaths were classified using the underlying cause of death data from the NDI and the *International Classification of Diseases*, Tenth Revision code (ICD-10 code C61). Vital status was ascertained from the Social Security Administration (SSA).

### Covariates: confounders and effect-measure modifiers

Based on previous literature and prior knowledge, we considered a variety of covariates that may influence the relationship between sleep characteristics and prostate cancer. These potential confounders include the following: age at enrollment (40–49, 50–59, 60–69, 70–79 years), enrollment source (community health center (CHC), general population), race/ethnicity (non-Hispanic White, non-Hispanic Black, other), educational attainment (less than 12 years, graduated high school/training/General Educational Development (GED), some college or junior college, graduated college or beyond), annual household income (< $15,000, $15,000–$24,999, $25,000–$49,999, ≥$50,000), body mass index (BMI) (< 18.5, 18.5-<25.0, 25.0- <30.0, ≥30.0 kg/m^2^), currently working (yes/no), smoking status (never, former, current), alcohol drinking status (non-drinkers, former drinkers, current drinkers), total physical activity (metabolic equivalent of task (MET)-hours/day-continuous), history of diabetes (yes/no), history of depression (yes/no), history of hypertension (yes/no), history of chronic obstructive pulmonary disease (COPD) (yes/no), history of asthma (yes/no), history of stroke (yes/no), and benign prostate hyperplasia (BPH) (yes/no). Race/ethnicity was used to assess effect-measure modification.

### Statistical analysis

To characterize the population, lifestyle, demographic, and socioeconomic variables were included. Means and standard deviations were presented for continuous variables, and frequencies and percentages were presented for categorical variables. To evaluate the association between sleep duration and restless sleep and prostate cancer-specific mortality in the analytic sample, multivariable Cox proportional hazards regression models were utilized to calculate hazard ratios and 95% confidence intervals to estimate mortality. The proportional hazards assumption was evaluated by Schoenfeld residuals and was considered met. In the Cox models, follow-up time was the time scale in calendar years. The participants were followed from the date of enrollment until the date of prostate cancer death or the end of follow-up (31 December 2022), whichever came first.

In our overall cohort analyses, we first fitted a minimally adjusted model that included age at enrollment and race/ethnicity, and then a fully adjusted model with additional covariates. For analyses stratified on race/ethnicity, we first fitted a minimally adjusted model of enrollment age and a fully adjusted model with covariates thereafter.

To assess effect modification by race/ethnicity, we stratified the cohort into two subgroups, namely non-Hispanic Black/African American and non-Hispanic White/Caucasian men, and evaluated the associations of sleep with prostate cancer-specific mortality in each subgroup. A Wald test was performed to assess the significance of the sleep–race/ethnicity interaction term.

All tests and models were conducted on non-missing data, and all variables included in the analyses had very low rates of unknown or missing data (ranging from 0.9 to 3.6%) and thus were handled via complete case analyses.

In the analyses investigating the association between sleep and prostate cancer-specific mortality and stratified by Black and White men, we also conducted sensitivity analyses by performing Fine and Gray’s sub-distribution hazard models to account for competing risks. Another sensitivity analysis we conducted was utilizing multiple imputation for missing data. We ran multiple imputations under the assumption that data were missing at random. Thirty complete datasets were generated, and 17 covariates were included for the imputation step, but the exposure variables were not imputed. Finally, another sensitivity analysis we conducted was to adjust four additional covariates in our fully adjusted models (Healthy Eating Index (HEI)− 2010 (continuous), family history of prostate cancer including father and brother (yes/no), prostate screening by prostate-specific antigen (PSA) test (yes/no), and digital rectal exam (DRE) (yes/no)).

Tests for trend for sleep were executed for sleep duration, and continuous P for trend was calculated by treating categorical sleep duration in the model as a continuous variable. All statistical significance tests were two-sided. All statistical analyses were performed using SAS statistical software (version 9.4; SAS Institute Inc., Cary, NC).

## Results

### Baseline and exposure characteristics 

During follow-up, 225 men developed fatal prostate cancer. The median follow-up time for the 33,568 male participants was 15.9  (interquartile range (IQR)= 6.1) years. Furthermore, the median weekday sleep duration was 7 h (IQR: 6.0–8.0) in both participants currently working and not working, and the median weekend sleep duration was 8 h (IQR: 6.0–8.0) in both participants currently working and not working. Table 1 shows selected baseline characteristics of the cohort and the men who died of prostate cancer and stratified by race/ethnicity subgroups. Overall, most men who died of prostate cancer were between 50 and 69 years of age at enrollment and had a lower household income (< $25,000), and about 45% of men reported being current smokers. Among Black men who died of prostate cancer, 60.5% of men reported having a household income of < $15,000, approximately half of the men reported being current smokers, and about 10–12% of men reported sleeping <6 h on average, on weekdays, and on weekends. Among White men who died of prostate cancer, the majority reported not currently working, and about half of the men reported experiencing restless sleep some of the time during the past week. Other baseline covariates that were used can be found in Supplemental Table S1a. Table 2 displays the characteristics of SCCS participants by weighted sleep average. Compared to the men who reported between 7 and 8 h of sleep, the short and long sleep duration groups were less likely to be currently working and more likely to be current smokers and have a history of depression.

**Table 1:** Baseline characteristics of men and prostate cancer (PC) deaths, overall and by race in the SCCS

	Full cohort (*n*=33,568)	Full cohort PC deaths (*n*=225)	Non-hispanic black men (*n*=22,437)	Non-hispanic black men PC deaths (*n*=178)	Non-hispanic white men (*n*=9,609)	Non-hispanic white men PC deaths (*n*=34)
**Baseline characteristic**	*N* (%)	*N* (%)	*N* (%)	*N* (%)	*N* (%)	*N* (%)
Enrollment age (years)						
40–49	15,478 (46.1)	42 (18.7)	11,304 (50.4)	40 (22.5)	3545 (36.9)	1 (2.9)
50–59	11,837 (35.3)	74 (32.9)	7092 (35.2)	63 (35.4)	3385 (35.2)	7 (20.6)
60–69	5002 (14.9)	76 (33.8)	2611 (11.6)	53 (29.8)	2114 (22.0)	19 (55.9)
70–79	1251 (3.7)	33 (14.7)	620 (2.8)	22 (12.4)	565 (5.9)	7 (20.6)
Annual household income						
< $15,000	17,714 (54.1)	120 (55.3)	13,151 (60.0)	104 (60.5)	3957 (42.0)	13 (39.4)
$15,000–$24,999	6565 (20.1)	48 (22.1)	4685 (21.4)	41 (23.8)	1603 (17.0)	5 (15.2)
$25,000–$49,999	4627 (14.1)	26 (12.0)	2761 (12.6)	17 (9.9)	1622 (17.2)	6 (18.2)
≥$50,000	3826 (11.7)	23 (10.6)	1331 (6.1)	10 (5.8)	2239 (23.8)	9 (27.3)
BMI (kg/m^2^)						
<18.5	386 (1.2)	4 (1.8)	284 (1.3)	3 (1.7)	85 (0.9)	0 (0.0)
18.5—<25.0	10,698 (32.5)	73 (33.5)	7698 (35.0)	59 (34.1)	2635 (27.4)	11 (32.4)
25.0 – <30.0	11,762 (35.8)	89 (40.8)	7699 (35.0)	70 (40.5)	3523 (37.1)	14 (41.2)
≥30.0	10,053 (30.6)	52 (23.9)	6313 (28.7)	41 (23.7)	3255 (34.3)	9 (26.5)
Currently working						
No	19,811 (60.3)	133 (60.7)	13,504 (61.5)	104 (59.8)	5537 (58.4)	24 (72.7)
Yes	13,057 (39.7)	86 (39.3)	8464 (38.5)	70 (40.2)	3951 (41.6)	9 (27.3)
Smoking status						
Never	7658 (23.3)	62 (28.3)	4893 (22.2)	46 (26.4)	2406 (25.5)	12 (35.3)
Former	8097 (24.7)	59 (26.9)	4543 (20.6)	44 (25.3)	3141 (33.3)	12 (35.3)
Current	17,060 (52.0)	98 (44.8)	12,577 (57.1)	84 (48.3)	3882 (41.2)	10 (29.4)
Alcohol drinking status						
Non-drinkers	2018 (6.2)	21 (9.6)	1243 (5.7)	11 (6.3)	671 (7.1)	6 (18.2)
Former drinkers	8308 (25.4)	62 (28.3)	5094 (23.3)	50 (28.7)	2846 (30.2)	9 (27.3)
Current drinkers	22,343 (68.4)	136 (62.1)	15,545 (71.0)	113 (64.9)	5901 (62.7)	18 (54.6)
History of depression						
No	27,506 (83.1)	200 (90.1)	19,327 (87.3)	164 (93.2)	7091 (74.3)	26 (76.5)
Yes	5604 (16.9)	22 (9.9)	2804 (12.7)	12 (6.8)	2456 (25.7)	8 (23.5)
BPH						
No	30,272 (91.9)	185 (83.3)	20,864 (94.6)	156 (88.6)	8157 (86.0)	23 (67.7)
Yes	2668 (8.1)	37 (16.7)	1181 (5.4)	20 (11.4)	1332 (14.0)	11 (32.4)
Sleep average (hours)						
<6	4661 (14.3)	25 (11.4)	3168 (14.5)	20 (11.6)	1254 (13.3)	4 (11.8)
6	6502 (19.9)	37 (16.9)	4231 (19.4)	29 (16.8)	1987 (21.0)	6 (17.7)
7–8	15,620 (47.9)	107 (48.9)	9916 (45.5)	81 (46.8)	5041 (53.4)	19 (55.9)
≥9	5858 (18.0)	50 (22.8)	4496 (20.6)	43 (24.9)	1164 (12.3)	5 (14.7)
Sleep weekday (hours)						
<6	5106 (15.6)	24 (10.9)	3467 (15.8)	19 (10.9)	1373 (14.5)	4 (11.8)
6	7889 (24.1)	44 (20.0)	5220 (23.8)	34 (19.5)	2332 (24.6)	8 (23.5)
7–8	15,394 (46.9)	112 (50.9)	9895 (45.2)	86 (49.4)	4852 (51.2)	18 (52.9)
≥9	4411 (13.5)	40 (18.2)	3335 (15.2)	35 (20.1)	926 (9.8)	4 (11.8)
Sleep weekend (hours)						
<6	4644 (14.2)	25 (11.4)	3218 (14.7)	20 (11.6)	1182 (12.5)	3 (8.8)
6	6223 (19.0)	43 (19.6)	4206 (19.2)	33 (19.1)	1769 (18.7)	8 (23.5)
7–8	14,529 (44.4)	90 (41.1)	9016 (41.2)	68 (39.3)	4894 (51.7)	16 (47.1)
≥9	7364 (22.5)	61 (27.9)	5450 (24.9)	52 (30.1)	1629 (17.2)	7 (20.6)
Restless sleep						
Rarely or none of the time	11,843 (36.2)	98 (44.8)	8301 (37.8)	82 (47.4)	3064 (32.5)	11 (32.4)
Some of the time	12,960 (39.6)	82 (37.4)	8929 (40.7)	63 (36.4)	3555 (37.7)	17 (50.0)
Much of the time	3726 (11.4)	11 (5.0)	2156 (9.8)	6 (3.5)	1369 (14.5)	1 (2.9)
Most or all of the time	4220 (12.9)	28 (12.8)	2551 (11.6)	22 (12.7)	1443 (15.3)	5 (14.7)

Adapted from [5]. Some percentages may not equal to 100.0 due to rounding. Missing data not included in percentages

*SCCS* Southern Community Cohort Study, *BMI* body mass index, *BPH* benign prostate hyperplasia

**Table 2:** Baseline characteristics of men by weighted sleep average in the SCCS, 2002–2009

	<6 Hours	6 Hours	7–8 Hours	≥9 Hours	*p*-value^a^
**Baseline characteristic**	*N* (%)	*N* (%)	*N* (%)	*N* (%)	
Enrollment age (years)					<.0001
40–49	2388 (51.2)	3079 (47.4)	6907 (44.2)	2787 (47.6)	
50–59	1690 (36.3)	2347 (36.1)	5472 (35.0)	1969 (33.6)	
60–69	480 (10.3)	885 (13.6)	2582 (16.5)	860 (14.7)	
70–79	103 (2.2)	191 (2.9)	659 (4.2)	242 (4.1)	
Annual household income					<.0001
< $15,000	2873 (62.4)	3290 (51.2)	7618 (49.4)	3689 (63.6)	
$15,000–$24,999	896 (19.4)	1372 (21.4)	3129 (20.3)	1085 (18.7)	
$25,000–$49,999	549 (11.9)	1015 (15.8)	2291 (14.9)	682 (11.8)	
≥$50,000	290 (6.3)	747 (11.6)	2375 (15.4)	345 (6.0)	
BMI (kg/m^2^)					<.0001
<18.5	71 (1.5)	79 (1.2)	150 (0.97)	76 (1.3)	
18.5–<25.0	1501 (32.5)	2003 (31.0)	5028 (32.4)	2046 (35.3)	
25.0–<30.0	1515 (32.8)	2319 (35.9)	5807 (37.5)	1930 (33.3)	
≥30.0	1531 (33.2)	2068 (32.0)	4514 (29.1)	1750 (30.2)	
Currently working					<.0001
No	3105 (67.1)	3701 (57.3)	8708 (56.1)	4038 (69.5)	
Yes	1524 (32.9)	2761 (42.7)	6818 (43.9)	1771 (30.5)	
Smoking status					<.0001
Never	967 (20.9)	1547 (24.0)	3855 (24.9)	1168 (20.1)	
Former	995 (21.5)	1553 (24.1)	4096 (26.5)	1285 (22.1)	
Current	2673 (57.7)	3355 (52.0)	7505 (48.6)	3360 (57.8)	
Alcohol drinking status					0.002
Non-drinkers	278 (6.0)	391 (6.1)	965 (6.3)	352 (6.1)	
Former drinkers	1236 (26.8)	1657 (25.8)	3755 (24.3)	1552 (26.8)	
Current drinkers	3095 (67.2)	4383 (68.2)	10,714 (69.4)	3890 (67.1)	
History of depression					<.0001
No	3376 (72.5)	5361 (82.5)	13,487 (86.4)	4868 (83.1)	
Yes	1281 (27.5)	1140 (17.5)	2127 (13.6)	987 (16.9)	
BPH					<.0001
No	4284 (92.4)	5939 (91.8)	14,182 (91.2)	5470 (93.8)	
Yes	355 (7.7)	534 (8.3)	1362 (8.8)	362 (6.2)	

Adapted from [5]. Some percentages may not equal to 100.0 due to rounding. Missing data not included in percentages.

*SCCS* Southern Community Cohort Study, *BMI* body mass index, *BPH* benign prostate hyperplasia

^a^*P*-values represent Pearson chi-square tests of association for categorical variables.

The missing data for each sleep trait of the overall study population was less than 3.6%, and the frequency and percentage of missing values in the exposure variables and covariates can be found in Supplemental Table S1b. Supplemental Tables S2a, S2b, and S2c show the mean weighted sleep average, weekday, and weekend sleep durations by restless sleep in the overall cohort and when stratified by race. Across the restless sleep categories, the mean sleep average, weekday, and weekend sleep durations differed significantly (*p* <0.0001). Furthermore, participants slept an average of 0.51 h longer on the weekends than on the weekdays (mean difference = 0.51 h; 95% CI 0.49–0.53). Supplemental Table S3 displays the correlations between weekday sleep and weekend sleep in the cohort and among race-specific groups. Baseline weekday and weekend sleep duration were positively correlated in the overall cohort (*r*=0.65) and among Non-Hispanic Black men (*r*=0.62) and moderately correlated among Non-Hispanic White men only (*r*=0.75).

Supplemental Table S4 showcases how the sleep categories changed from baseline average sleep duration to follow-up 1 overall sleep duration. Among 16,484 participants with both baseline sleep average and follow-up 1 sleep information, there was consistency in reported sleep duration between the two time points, with the majority of participants remaining in the same sleep category. For example, among participants sleeping 7–8 h at baseline, ~63% reported sleeping between 7 and 8 h at follow-up 1.

### Overall prostate cancer-specific mortality and stratified by race/ethnicity

Tables 3 and 4 show the hazard ratios for associations of sleep and prostate cancer-specific mortality, and when stratified by race/ethnicity. In the analysis of the entire cohort, we did not observe a consistent association between overall prostate cancer-specific mortality and weighted sleep average duration and weekday sleep duration, or restless sleep. However, long sleep duration (≥ 9 h/day) on weekends was associated with a 44% (HR=1.44, 95% CI 1.02–2.03) increased risk of prostate cancer-specific mortality compared to 7–8 h/day, although there was a limited number of prostate cancer deaths for that category (Table 3).

**Table 3:** Associations between sleep characteristics at enrollment and prostate cancer-specific mortality in the SCCS

	Cohort *n*=33,568	Deaths *n*=225	Age and race/ethnicity adjusted^a^	Fully adjusted^b^
	*N* (%)^c^	*N* (%)^c^	HR (95% CI)	HR (95% CI)
Sleep duration, hr				
Sleep average				
<6	4388 (14.4)	22 (11.2)	0.91 (0.58–1.42)	0.92 (0.58–1.47)
6	6094 (20.0)	34 (17.3)	0.90 (0.62–1.30)	0.91 (0.62–1.35)
7–8	14,528 (47.7)	95 (48.2)	1.00 (Ref)	1.00 (Ref)
≥9	5480 (18.0)	46 (23.4)	1.31 (0.93–1.83)	1.35 (0.94–1.93)
			*p trend*=*0.10*	*p trend*=*0.11*
Weekday				
<6	4785 (15.7)	21 (10.6)	0.76 (0.48–1.19)	0.77 (0.48–1.23)
6	7394 (24.2)	39 (19.7)	0.85 (0.60–1.21)	0.85 (0.58–1.23)
7–8	14,278 (46.7)	100 (50.5)	1.00 (Ref)	1.00 (Ref)
≥9	4108 (13.4)	38 (19.2)	1.26 (0.88–1.82)	1.34 (0.92–1.95)
			*p trend*=*0.03*	*p trend*=*0.03*
Weekend				
<6	4382 (14.3)	20 (10.2)	1.05 (0.67–1.65)	0.92 (0.56–1.50)
6	5850 (19.1)	41 (20.8)	1.23 (0.85–1.77)	1.25 (0.85–1.82)
7–8	13,482 (44.1)	81 (41.1)	1.00 (Ref)	1.00 (Ref)
≥9	6847 (22.4)	55 (27.9)	1.44 (1.04–2.00)	1.44 (1.02–2.03)
			*p trend*= *0.29*	*p trend*= *0.18*
Restless sleep				
Rarely/none of the time	11,057 (36.1)	87 (44.2)	1.00 (Ref)	1.00 (Ref)
Some of the time	12,114 (39.6)	75 (38.1)	0.88 (0.66–1.18)	0.92 (0.67–1.26)
Much of the time	3470 (11.3)	10 (5.1)	0.55 (0.29–1.02)	0.57 (0.29–1.11)
Most/all of the time	3955 (12.9)	25 (12.7)	1.18 (0.77–1.82)	1.22 (0.77–1.94)

Of the 33,568 participants in the analytic cohort, 2,848 were excluded from the fully adjusted models for each exposure due to any missing covariate data. Multiple comparisons were adjusted using the Bonferroni method, and results were not significant

*HR* hazard ratio; *95% CI* 95% confidence interval

^a^HR was adjusted for age at enrollment and race/ethnicity

^b^HR was additionally adjusted for enrollment source, education, income, employment status, BMI, depression, alcohol drinking, smoking status, diabetes, hypertension, COPD, asthma, stroke, total physical activity, and BPH

^c^The cohort and death numbers reflect the fully adjusted model numbers

**Table 4:** Associations between sleep characteristics at enrollment and prostate cancer-specific mortality by race/ethnicity in the SCCS

	Deaths (Black) *n*=178	Deaths (White) *n*=34	Age-adjusted^a^		Race/ethnicity-specific (fully adjusted)^b^		
			Non-Hispanic Black	Non-Hispanic White	Non-Hispanic Black	Non-Hispanic White	*p* interaction
	*N* (%)^c^	*N* (%)^c^	HR (95% CI)	HR (95% CI)	HR (95% CI)	HR (95% CI)	
**Sleep duration, hr**							
** Sleep average**							
<6	18 (11.4)	4 (13.8)	0.88 (0.54–1.44)	1.86 (0.63–5.54)	0.89 (0.53–1.50)	1.70 (0.53–5.48)	
6	26 (16.5)	6 (20.7)	0.88 (0.58–1.35)	1.04 (0.41–2.61)	0.88 (0.56–1.38)	1.27 (0.48–3.37)	
7–8	74 (46.8)	14 (48.3)	1.00 (Ref)	1.00 (Ref)	1.00 (Ref)	1.00 (Ref)	
≥9	40 (25.3)	5 (17.2)	1.29 (0.89–1.87)	1.34 (0.50–3.59)	1.30 (0.88–1.91)	1.79 (0.63–5.15)	*0.53*
Weekday			*p trend*=*0.10*	*p trend*=*0.66*	*p trend*=*0.13*	*p trend*=*0.86*	
<6	17 (10.7)	4 (13.8)	0.72 (0.44–1.19)	1.74 (0.58–5.20)	0.74 (0.43–1.25)	1.60 (0.50–5.13)	
6	30 (18.9)	7 (24.1)	0.81 (0.55–1.21)	1.27 (0.55–2.93)	0.82 (0.53–1.25)	1.37 (0.54–3.46)	
7–8	78 (49.1)	14 (48.3)	1.00 (Ref)	1.00 (Ref)	1.00 (Ref)	1.00 (Ref)	
≥9	34 (21.4)	4 (13.8)	1.28 (0.87–1.90)	1.29 (0.44–3.82)	1.34 (0.89–2.01)	1.71 (0.55–5.38)	*0.35*
Weekend			*p trend*=*0.02*	*p trend*=*0.49*	*p trend*=*0.03*	*p trend*=*0.74*	
<6	16 (10.1)	3 (10.3)	0.99 (0.60–1.64)	1.84 (0.53–6.39)	0.84 (0.49–1.46)	1.69 (0.44–6.47)	
6	31 (19.6)	8 (27.6)	1.15 (0.76–1.74)	1.80 (0.77–4.21)	1.14 (0.74–1.76)	2.24 (0.88–5.69)	
7–8	64 (40.5)	11 (37.9)	1.00 (Ref)	1.00 (Ref)	1.00 (Ref)	1.00 (Ref)	
≥9	47 (29.8)	7 (24.1)	1.42 (0.99–2.04)	1.65 (0.68–4.01)	1.36 (0.93–1.99)	2.57 (0.98–6.75)	*0.63*
			*p trend*=*0.21*	*p trend*=*0.57*	*p trend*=*0.15*	*p trend*=*1.00*	
Restless sleep							
Rarely/none of the time	74 (46.8)	9 (31.0)	1.00 (Ref)	1.00 (Ref)	1.00 (Ref)	1.00 (Ref)	
Some of the time	59 (37.3)	14 (48.3)	0.82 (0.59–1.14)	1.50 (0.70–3.19)	0.84 (0.59–1.19)	1.64 (0.70–3.83)	
Much of the time	5 (3.2)	1 (3.5)	0.37 (0.16–0.86)	0.37 (0.05–2.89)	0.36 (0.14–0.89)	0.41 (0.05–3.30)	
Most/all of the time	20 (12.7)	5 (17.2)	1.17 (0.73–1.88)	2.01 (0.69–5.88)	1.21 (0.73–2.02)	2.02 (0.63–6.52)	*0.39*

Among those who are black and white only (*n*=32,046). Due to small sample size, “Other” race (*n*=1215) was excluded. Analysis is complete case only (thus observations with missing covariates or exposure of interest were excluded). Of the 22,437 black participants and 9,609 white participants in the analytic cohort, 1,567 were excluded from the fully adjusted models for each exposure due to any missing covariate data for black men, and 850 were excluded from the fully adjusted models for each exposure due to any missing covariate data for white men. Multiple comparisons were adjusted using the Bonferroni method, and results were not significant

*HR* hazard ratio; *95% CI* 95% confidence interval

^a^ HR was adjusted for age at enrollment

^b^ HR was additionally adjusted for enrollment source, education, income, employment status, BMI, depression, alcohol drinking, smoking status, diabetes, hypertension, COPD, asthma, stroke, total physical activity, and BPH

^c^ The death numbers reflect the fully adjusted model numbers

In the analyses stratified by Black and White men separately, there were no consistent associations between sleep duration and prostate cancer-specific mortality among either Black or White men. There was no clear interaction between sleep duration (and restless sleep) and race/ethnicity in relation to prostate cancer-specific mortality. However, we did observe that there was some supportive evidence for an increased risk in prostate cancer-specific mortality for long duration (≥ 9 h/day) for weekend sleep duration in comparison to 7–8 h/day for both Black men (HR= 1.36, 95% CI 0.93–1.99) and White men (HR= 2.57, 95% CI 0.98–6.75), although the confidence interval for both Black and White men included the null and small number of prostate cancer deaths (Table 4).

### Sensitivity analyses

Supplemental Table S5 showcases results when we estimated sub-distribution hazard ratios (SHRs) via Fine and Gray models for the overall cohort and when stratified by Black/White men, respectively (Supplemental Table S6). Results were unchanged in comparison to our main analyses, and there was limited influence of competing risks of death on prostate cancer mortality. Supplemental Table S7 consists of multiple imputations of covariates for prostate cancer-specific mortality for the whole cohort, and when stratified by race/ethnicity (Supplemental Table S8). Results with multiple imputations yielded similar results, compared with complete case analyses. When we additionally adjusted for PSA screening, DRE screening, HEI-2010, and family history of prostate cancer in our fully adjusted models, the inferences were consistent for prostate cancer-specific mortality and when stratified by Black and White men (Supplemental Tables S9 and S10). Finally, we adjusted for age as a continuous variable as a sensitivity analysis, and the associations observed were still consistent [data not shown].

## Discussion

In this cohort consisting of 33,568 men with 15.9 (median) years of follow-up, we did not observe a clear association between sleep characteristics and prostate cancer-specific mortality in the overall cohort. While we hypothesized that there would be a positive association between shorter sleep duration and prostate cancer-specific mortality, in our study, we found no clear associations. However, we did observe that long sleep duration on weekends, in comparison to optimal sleep duration (7–8 h/day), was suggestively associated with an increased risk of prostate cancer-specific mortality, although limited to small numbers. When stratified by Black and White men only, we saw similar associations of increased risk for longer sleep duration for sleep weekend for both races, although based on small numbers of deaths and some 95% confidence levels including the null. There was no clear evidence of interactions of sleep characteristics with race/ethnicity.

The biological mechanisms relating to the potential associations between longer sleep duration and prostate cancer mortality are not fully understood and are complex. One possible explanation is that long sleep duration could be the result of poor health rather than a direct cause in cancer risk [1]. Sleep may be tapping into an underlying health status that is driving prostate cancer-specific mortality. How well people sleep and how long they sleep could just be a useful surrogate or proxy of an underlying health condition such as depression or hypertension. Moreover, longer sleep duration on weekends could be due to irregular sleep during the weekdays. Taylor et al*.* found that sleeping in late on weekends had a circadian rhythm delay and fatigue and increased daytime sleepiness the following weekdays [18].

In our study, we found that long sleep duration was tentatively associated with prostate cancer-specific mortality for both Black and White men, although the number of mortality events for sleeping ≥ 9 h was small (especially for White men), and no racial differences were found. The biological mechanisms for such associations are unclear. There may be other risk factors such as occupational factors or physical environments that may affect sleep for both Black and White men which could have led to mortality. Furthermore, circadian rhythm disruption (CRD) has been suggested to be associated with prostate cancer. African American men may be more likely to develop CRD [19]. African American men are more susceptible to CRD due to a variety of reasons, including racial discrimination, environmental factors, socioeconomic disadvantages, and exposure to night shift work [19]. Consequences of CRD might be associated with behavioral stress and psychosocial aspects of health, eating disorders, obesity, and sleep deprivation. African American men are disproportionately affected by these consequences which in turn can worsen racial disparities [20]. Very few studies focused on sleep and prostate cancer-specific mortality; however, our findings for sleep duration and sleep quality were in line with some [7–11], but not all [12]. For example, in a Finnish twin cohort study, Dickerman et al*.* found no significant association between sleep duration or quality and prostate cancer-specific mortality, but there was a suggestive positive association, although imprecise and confidence intervals crossing the null, between longer sleep duration and mortality [(HR=1.5, 95% CI 0.8–2.8 for >8 h vs. ref (< 7 h))] [10]. In a large prospective cohort study of U.S. adults, however, Gapstur et al. concluded that short sleep duration was associated with an increased risk of fatal prostate cancer during the first 8 years of follow-up [relative risks (RR=1.64, 95% CI 1.06–2.54 for 3–5 h vs. ref (7 h) and RR= 1.28, 95% CI 0.98–1.67 for 6 h vs. ref (7 h))] [12]. The prior studies did not address the race/ethnicity component as their populations were majority White. To our knowledge, this is one of the first studies to examine self-reported sleep duration and restless sleep and prostate cancer-specific mortality among a low-income and predominantly Non-Hispanic Black/African American cohort.

This study had many strengths. It was based on a large-scale prospective cohort, had a long-term follow-up, and we were able to adjust for a wide range of potential confounders based on detailed baseline information. Moreover, our study was diverse and consisted of predominantly Black/African American men, who are disproportionately affected by prostate cancer mortality. Despite its strengths, there were also some potential limitations. One limitation is that we focused on a single self-reported measure of sleep duration, which may not reflect participants’ long-term sleep patterns. Second, we lacked information on other sleep characteristics, such as sleep medication use, sleep disorders, shift work, snoring, chronotype, and medical treatments that may affect sleep. Assessing additional sleep characteristics could have provided a more comprehensive understanding of the association between sleep and prostate cancer mortality. Third, our sleep exposure variables were self-reported, which could have resulted in exposure misclassification, with some participants reporting short sleep or long sleep being misclassified as normal sleep, or vice versa. Finally, the number of prostate cancer deaths was relatively small, particularly within the extreme categories of sleep duration. Consequently, statistical power was limited, which may have reduced the precision of hazard ratio estimates and our ability to detect statistically significant associations. Future studies with a larger number of events are warranted to investigate the association between sleep and prostate cancer-specific mortality.

## Conclusion

In conclusion, while we did not see clear associations between sleep duration and restless sleep and prostate cancer mortality, there were suggestive positive associations of an increased risk of prostate cancer-specific mortality that was observed with some sleep characteristics, including longer sleep duration on weekends in both Black and White men, although underpowered. Our study is one of the first studies that has looked at self-reported sleep indicators and prostate cancer-specific mortality among Black men. Future studies with information on other sleep characteristics, such as sleep medications and sleep disorders, and conducting in a diverse population are crucial to exploring the associations between sleep and prostate cancer-specific mortality.

## Supplementary Information

Below is the link to the electronic supplementary material.

Supplementary file1 (DOCX 77 KB)

## Data Availability

The data underlying this article were obtained through a data access agreement with the Southern Community Cohort Study. The analytic code, code book, and data are available from the corresponding author on reasonable request and in agreement with data access rules set by the Southern Community Cohort Study (https://ors.southerncommunitystudy.org/).

## Abbreviations

BPH: Benign prostate hyperplasia
BMI: Body mass index
COPD: Chronic obstructive pulmonary disease
CRD: Circadian rhythm disruption
CHC: Community health center
CI: Confidence interval
DRE: Digital rectal exam
GED: General Educational Development
HR: Hazard ratio
HEI: Healthy eating index
ICD: International Classification of Diseases
IQR: Interquartile range
MET: Metabolic equivalent of task
NDI: National Death Index
PSA: Prostate-specific antigen
SSA: Social Security Administration
SCCS: Southern Community Cohort Study
SHR: Sub-distribution hazard ratio
