# Supplementary material for: Sleep duration and prostate cancer-specific mortality in the Southern Community Cohort Study
Source: Cancer Causes Control. 2026 Oct 21;37(11):182. doi: 10.1007/s10552-026-02256-8 (PMC13687344; doi:10.1007/s10552-026-02256-8)
Supplement: Supplementary file 1 [file 10552_2026_2256_MOESM1_ESM.docx]

**Sleep Duration and Prostate Cancer-Specific Mortality in the Southern Community Cohort Study**

**Running title: Sleep and Prostate Cancer-Specific Mortality**

**Journal:** *Cancer Causes & Control*

Danica C. Anukam^1^, Roch A. Nianogo^1^, Onyebuchi A. Arah^1,2,3^, Jianyu Rao^1,4^, Jay H. Fowke^5^, Mia Hashibe^1,6^ , Zuo-Feng Zhang^1,6,^

Affiliations:

^1^ Department of Epidemiology, UCLA Fielding School of Public Health, Los Angeles, CA, USA

^2^ Department of Statistics and Data Science, UCLA College of Letters and Science, Los Angeles, CA, USA

^3^ Department of Public Health, Aarhus University, Aarhus, Denmark

^4^ Department of Pathology and Laboratory Medicine, UCLA David Geffen School of Medicine, Los Angeles, CA, USA

^5^ Department of Preventive Medicine, The University of Tennessee Health Science Center, College of Medicine, Memphis, TN, USA

^6^ Jonsson Comprehensive Cancer Center, UCLA, Los Angeles, CA, USA

**Corresponding Author:**

Zuo-Feng Zhang, MD, PhD

Department of Epidemiology

UCLA Fielding School of Public Health

650 Charles E Young Dr.

Los Angeles, CA 90095

Email: [zfzhang@ucla.edu](mailto:zfzhang@ucla.edu)

# **Supplemental Tables for Manuscript**

## **Table S1a. Additional baseline characteristics of prostate cancer deaths (PC), overall and by race in the SCCS**

|  | **Full Cohort PC Deaths (n=225)** | **Non-Hispanic Black Men PC Deaths (n=178)** | **Non-Hispanic White Men PC Deaths (n=34)** |
| --- | --- | --- | --- |
| **Baseline Characteristic** | **N (%)** | **N (%)** | **N (%)** |
| Enrollment source |  |  |  |
| CHC in-person interview | 193 (85.8) | 164 (92.1) | 22 (64.7) |
| General population | 32 (14.2) | 14 (7.9) | 12 (35.3) |
| Race/Ethnicity |  |  |  |
| Non-Hispanic White | 34 (15.3) | 0 (0.0) | 34 (100.0) |
| Non-Hispanic Black | 178 (79.8) | 178 (100.0) | 0 (0.0) |
| Other | 11 (4.9) | 0 (0.0) | 0 (0.0) |
| Educational attainment |  |  |  |
| Less than 12 years | 90 (40.7) | 84 (47.7) | 5 (14.7) |
| Graduated high school, training, or GED | 70 (31.7) | 56 (31.8) | 11 (32.4) |
| Some college or junior college | 37 (16.7) | 24 (13.6) | 9 (26.5) |
| Graduated college or beyond | 24 (10.9) | 12 (6.8) | 9 (26.5) |
| Total physical activity (MET-hours/day), mean ± SD | 23.3 ± 21.0 | 23.9 ± 21.6 | 15.9 ± 12.9 |
| History of diabetes |  |  |  |
| No | 187 (84.6) | 147 (84.0) | 30 (88.2) |
| Yes | 34 (15.4) | 28 (16.0) | 4 (11.8) |
| History of hypertension |  |  |  |
| No | 108 (48.7) | 88 (50.0) | 14 (41.2) |
| Yes | 114 (51.4) | 88 (50.0) | 20 (58.8) |
| History of COPD |  |  |  |
| No | 213 (96.0) | 171 (97.2) | 31 (91.2) |
| Yes | 9 (4.1) | 5 (2.8) | 3 (8.8) |
| History of asthma |  |  |  |
| No | 201 (90.5) | 157 (89.2) | 32 (94.1) |
| Yes | 21 (9.5) | 19 (10.8) | 2 (5.9) |
| History of stroke |  |  |  |
| No | 213 (96.0) | 169 (96.0) | 33 (97.1) |
| Yes | 9 (4.1) | 7 (4.0) | 1 (2.9) |
| HEI-2010, mean ± SD | 56.3 ± 11.1 | 56.0 ± 13.3 | 55.9 ± 10.2 |
| Family history of PC |  |  |  |
| No | 196 (94.7) | 156 (96.3) | 31 (91.2) |
| Yes | 11 (5.3) | 6 (3.7) | 3 (8.8) |
| PSA Screening |  |  |  |
| No | 91 (43.8) | 76 (45.8) | 10 (31.3) |
| Yes | 117 (56.3) | 90 (54.2) | 22 (68.8) |
| DRE Screening |  |  |  |
| No | 74 (33.8) | 61 (35.3) | 10 (29.4) |
| Yes | 145 (66.2) | 112 (64.7) | 24 (70.6) |

Note: Some percentages may not equal to 100.0 due to rounding. Missing data not included in percentages.

SCCS: Southern Community Cohort Study. CHC: Community Health Center. SD: standard deviation.

MET: metabolic equivalent of task. HEI-2010: Healthy Eating Index. COPD: Chronic obstructive

pulmonary disease. PC: prostate cancer. PSA: prostate cancer-specific antigen. DRE: digital rectal examination.

## Table S1b: Frequency and percentage of missing values in the variables of interest. Adapted from [1].

| **Variable** | **Frequency of missing values** | **% of missing values** |  |  |
| --- | --- | --- | --- | --- |
| **Sleep Average** | 927 | 2.8 |  |  |
| **Sleep Weekday** | 768 | 2.3 |  |  |
| **Sleep Weekend** | 808 | 2.4 |  |  |
| **Restless Sleep** | 819 | 2.4 |  |  |
| **Enrollment Age** | 0 | 0 |  |  |
| **Enrollment Source** | 0 | 0 |  |  |
| **Race/Ethnicity** | 307 | 0.9 |  |  |
| **Education** | 646 | 1.9 |  |  |
| **Household Income** | 836 | 2.5 |  |  |
| **Employed** | 700 | 2.1 |  |  |
| **BMI** | 669 | 2.0 |  |  |
| **BPH** | 628 | 1.9 |  |  |
| **Depression** | 458 | 1.4 |  |  |
| **Hypertension** | 450 | 1.3 |  |  |
| **COPD** | 468 | 1.4 |  |  |
| **Asthma** | 455 | 1.4 |  |  |
| **Stroke** | 473 | 1.4 |  |  |
| **Alcohol Drinking** | 899 | 2.7 |  |  |
| **Smoking Status** | 753 | 2.2 |  |  |
| **Diabetes** | 459 | 1.4 |  |  |
| **Physical Activity** | 1212 | 3.6 |  |  |
| **Diet (HEI-2010)** | 2816 | 8.4 |  |  |
| **Family History of PC** | 2185 | 6.5 |  |  |
| **PSA Screening** | 2386 | 7.1 |  |  |
| **DRE Screening** | 660 | 2.0 |  |  |

Missingness of exposure variables and covariates. Note: The last four covariates listed above pertained to sensitivity analyses.

## **Table S2a. Mean weighted sleep average, weekday, and weekend sleep durations according to restless sleep categories (Full Cohort)**

|  | **Sleep Average** | **Sleep Weekday** | **Sleep Weekend** |
| --- | --- | --- | --- |
| **Restless Sleep** | Mean ± SD | Mean ± SD | Mean ± SD |
| Rarely/none of the time | 7.7 ± 1.8 | 7.6 ± 1.8 | 8.1 ± 2.3 |
| Some of the time | 7.4 ± 1.8 | 7.2 ± 1.9 | 7.8 ± 2.5 |
| Much of the time | 6.8 ± 2.0 | 6.6 ± 2.0 | 7.2 ± 2.5 |
| Most/all of the time | 6.2 ± 2.4 | 6.1 ± 2.4 | 6.5 ± 2.8 |

SD: standard deviation.

## **Table S2b. Mean weighted sleep average, weekday, and weekend sleep durations according to restless sleep categories (Non-Hispanic Black Men)**

|  | **Sleep Average** | **Sleep Weekday** | **Sleep Weekend** |
| --- | --- | --- | --- |
| **Restless Sleep** | Mean ± SD | Mean ± SD | Mean ± SD |
| Rarely/none of the time | 7.8 ± 1.9 | 7.6 ± 1.9 | 8.2 ± 2.5 |
| Some of the time | 7.4 ± 2.0 | 7.3 ± 2.0 | 7.9 ± 2.8 |
| Much of the time | 6.8 ± 2.1 | 6.6 ± 2.2 | 7.2 ± 2.7 |
| Most/all of the time | 6.4 ± 2.5 | 6.3 ± 2.5 | 6.7 ± 3.0 |

SD: standard deviation.

## **Table S2c. Mean weighted sleep average, weekday, and weekend sleep durations according to restless sleep categories (Non-Hispanic White Men)**

|  | **Sleep Average** | **Sleep Weekday** | **Sleep Weekend** |
| --- | --- | --- | --- |
| **Restless Sleep** | Mean ± SD | Mean ± SD | Mean ± SD |
| Rarely/none of the time | 7.5 ± 1.4 | 7.4 ± 1.4 | 7.8 ± 1.7 |
| Some of the time | 7.3 ± 1.5 | 7.1 ± 1.5 | 7.6 ± 1.8 |
| Much of the time | 6.7 ± 1.7 | 6.6 ± 1.8 | 7.1 ± 2.0 |
| Most/all of the time | 6.0 ± 2.3 | 5.9 ± 2.3 | 6.2 ± 2.4 |

SD: standard deviation.

## **Table S3. Correlations of sleep weekday and sleep weekend, overall and by race**

|  | **Full Cohort** | **Non-Hispanic Black Men** | **Non-Hispanic White Men** |
| --- | --- | --- | --- |
| **Correlation of Sleep Weekday and Weekend (Pearson r)** | 0.65 | 0.62 | 0.75 |

## **Table S4. Proportions of baseline sleep average and overall sleep duration at follow-up 1**

| **Sleep average (baseline), hours. n (%)** | **Overall sleep (follow-up 1), hours. n (%)** | | | | **Total** |
| --- | --- | --- | --- | --- | --- |
|  |  |  |  |  |  |
|  | **<6** | **6** | **7 to 8** | **≥9** |  |
| **<6** | 680 (31.14%) | 583 (26.69%) | 643 (29.44%) | 278 (12.73%) | 2184 (13.25%) |
| **6** | 476 (14.16%) | 1041 (30.97%) | 1429 (42.52%) | 415 (12.35%) | 3361 (20.39%) |
| **7 to 8** | 552 (6.65%) | 1255 (15.13%) | 5219 (62.90%) | 1271 (15.32%) | 8297 (50.33%) |
| **≥9** | 189 (7.15%) | 282 (10.67%) | 1249 (47.27%) | 922 (34.90%) | 2642 (16.03%) |

## **Table S5. Complete Analyses: Associations between sleep characteristics at enrollment and prostate cancer-specific mortality via Fine and Gray in the SCCS**

|  | Cohort  N=33,568 | Deaths  N=225 | Age and race/ethnicity adjusted^a^ | Fully adjusted^b^ |
| --- | --- | --- | --- | --- |
|  | Overall | Overall | Overall | Overall |
|  | N (%)^c^ | N (%)^c^ | HR (95% CI) | HR (95% CI) |
| Sleep duration, hr |  |  |  |  |
| Sleep average |  |  |  |  |
| <6 | 4388 (14.4) | 22 (11.2) | 0.84 (0.54-1.31) | 0.90 (0.56-1.45) |
| 6 | 6094 (20.0) | 34 (17.3) | 0.89 (0.61-1.30) | 0.93 (0.63-1.38) |
| 7-8 | 14528 (47.7) | 95 (48.2) | 1.00 (Ref) | 1.00 (Ref) |
| ≥9 | 5480 (18.0) | 46 (23.4) | 1.16 (0.83-1.63) | 1.30 (0.91-1.86) |
|  |  |  | *p trend=0.14* | *p trend=0.14* |
|  |  |  |  |  |
| Weekday |  |  |  |  |
| <6 | 4785 (15.7) | 21 (10.6) | 0.71 (0.45-1.11) | 0.75 (0.46-1.21) |
| 6 | 7394 (24.2) | 39 (19.7) | 0.85 (0.60-1.21) | 0.85 (0.58-1.24) |
| 7-8 | 14278 (46.7) | 100 (50.5) | 1.00 (Ref) | 1.00 (Ref) |
| ≥9 | 4108 (13.4) | 38 (19.2) | 1.12 (0.78-1.61) | 1.30 (0.89-1.89) |
|  |  |  | *p trend=0.05* | *p trend=0.03* |
|  |  |  |  |  |
| Weekend |  |  |  |  |
| <6 | 4382 (14.3) | 20 (10.2) | 0.96 (0.61-1.50) | 0.90 (0.55-1.49) |
| 6 | 5850 (19.1) | 41 (20.8) | 1.20 (0.83-1.74) | 1.27 (0.87-1.87) |
| 7-8 | 13482 (44.1) | 81 (41.1) | 1.00 (Ref) | 1.00 (Ref) |
| ≥9 | 6847 (22.4) | 55 (27.9) | 1.33 (0.96-1.85) | 1.40 (0.99-1.98) |
|  |  |  | *p trend= 0.31* | *p trend= 0.24* |
|  |  |  |  |  |
| Restless sleep |  |  |  |  |
| Rarely/none of the time | 11057 (36.1) | 87 (44.2) | 1.00 (Ref) | 1.00 (Ref) |
| Some of the time | 12114 (39.6) | 75 (38.1) | 0.90 (0.67-1.20) | 0.96 (0.70-1.31) |
| Much of the time | 3470 (11.3) | 10 (5.1) | 0.51 (0.28-0.96) | 0.57 (0.30-1.10) |
| Most/all of the time | 3955 (12.9) | 25 (12.7) | 1.07 (0.70-1.64) | 1.22 (0.76-1.94) |

HR: hazard ratio; 95% CI: 95% confidence interval

^a^ HR was adjusted for age at enrollment and race/ethnicity

^b^ HR was additionally adjusted for enrollment source, education, income, employment status, BMI, depression, alcohol drinking, smoking status, diabetes, hypertension, COPD, asthma, stroke, total physical activity, and BPH.

^c^ The cohort and death numbers reflect the fully adjusted model numbers.

## **Table S6. Complete Analyses: Associations between sleep characteristics at enrollment and prostate cancer-specific mortality by race via Fine and Gray in the SCCS (Cohort N=32,046)**

|  | Deaths N (Black) =178 | Deaths N (White) =34 | Age-adjusted^a^ | |  | Race/ethnicity-specific (fully adjusted)^b^ | | |
| --- | --- | --- | --- | --- | --- | --- | --- | --- |
|  |  |  | **Non-Hispanic Black** | **Non-Hispanic White** |  | **Non-Hispanic Black** | **Non-Hispanic White** | **p interaction** |
|  | N (%)^c^ | N (%)^c^ | HR (95% CI) | HR (95% CI) |  | HR (95% CI) | HR (95% CI) |  |
| Sleep duration, hr |  |  |  |  |  |  |  |  |
| Sleep average |  |  |  |  |  |  |  |  |
| <6 | 18 (11.4) | 4 (13.8) | 0.84 (0.52-1.37) | 1.44 (0.50-4.15) |  | 0.90 (0.53-1.52) | 1.66 (0.53-5.20) |  |
| 6 | 26 (16.5) | 6 (20.7) | 0.88 (0.58-1.35) | 1.00 (0.39-2.53) |  | 0.90 (0.58-1.41) | 1.26 (0.47-3.38) |  |
| 7-8 | 74 (46.8) | 14 (48.3) | 1.00 (Ref) | 1.00 (Ref) |  | 1.00 (Ref) | 1.00 (Ref) |  |
| ≥9 | 40 (25.3) | 5 (17.2) | 1.18 (0.82-1.71) | 1.09 (0.41-2.91) |  | 1.28 (0.86-1.90) | 1.58 (0.57-4.36) | *0.57* |
|  |  |  | *p trend=0.16* | *p trend=0.74* |  | *p trend=0.17* | *p trend=0.81* |  |
|  |  |  |  |  |  |  |  |  |
| Weekday |  |  |  |  |  |  |  |  |
| <6 | 17 (10.7) | 4 (13.8) | 0.70 (0.43-1.15) | 1.38 (0.48-3.97) |  | 0.74 (0.43-1.26) | 1.53 (0.50-4.75) |  |
| 6 | 30 (18.9) | 7 (24.1) | 0.82 (0.55-1.22) | 1.23 (0.53-2.86) |  | 0.82 (0.53-1.26) | 1.34 (0.53-3.38) |  |
| 7-8 | 78 (49.1) | 14 (48.3) | 1.00 (Ref) | 1.00 (Ref) |  | 1.00 (Ref) | 1.00 (Ref) |  |
| ≥9 | 34 (21.4) | 4 (13.8) | 1.17 (0.79-1.73) | 1.04 (0.35-3.08) |  | 1.33 (0.89-1.99) | 1.45 (0.49-4.32) | *0.38* |
|  |  |  | *p trend=0.04* | *p trend=0.55* |  | *p trend=0.03* | *p trend=0.70* |  |
|  |  |  |  |  |  |  |  |  |
| Weekend |  |  |  |  |  |  |  |  |
| <6 | 16 (10.1) | 3 (10.3) | 0.95 (0.58-1.56) | 1.35 (0.40-4.54) |  | 0.85 (0.49-1.48) | 1.65 (0.45-6.02) |  |
| 6 | 31 (19.6) | 8 (27.6) | 1.14 (0.75-1.73) | 1.73 (0.74-4.05) |  | 1.16 (0.76-1.79) | 2.32 (0.91-5.91) |  |
| 7-8 | 64 (40.5) | 11 (37.9) | 1.00 (Ref) | 1.00 (Ref) |  | 1.00 (Ref) | 1.00 (Ref) |  |
| ≥9 | 47 (29.8) | 7 (24.1) | 1.35 (0.94-1.93) | 1.40 (0.58-3.41) |  | 1.35 (0.92-1.97) | 2.27 (0.90-5.74) | *0.63* |
|  |  |  | *p trend=0.25* | *p trend=0.69* |  | *p trend=0.18* | *p trend=0.89* |  |
|  |  |  |  |  |  |  |  |  |
| continued | **Deaths N (Black) =178** | **Deaths N (White) =34** | **Age-adjusted^a^** | |  | **Race/ethnicity-specific (fully adjusted)^b^** | | |
|  |  |  | **Non-Hispanic Black** | **Non-Hispanic White** |  | **Non-Hispanic Black** | **Non-Hispanic White** | **p interaction** |
|  | N (%)^c^ | N (%)^c^ | HR (95% CI) | HR (95% CI) |  | HR (95% CI) | HR (95% CI) |  |
| Restless sleep |  |  |  |  |  |  |  |  |
| Rarely/none of the time | 74 (46.8) | 9 (31.0) | 1.00 (Ref) | 1.00 (Ref) |  | 1.00 (Ref) | 1.00 (Ref) |  |
| Some of the time | 59 (37.3) | 14 (48.3) | 0.83 (0.59-1.15) | 1.54 (0.71-3.32) |  | 0.88 (0.62-1.25) | 1.66 (0.70-3.94) |  |
| Much of the time | 5 (3.2) | 1 (3.5) | 0.36 (0.16-0.82) | 0.33 (0.04-2.63) |  | 0.36 (0.15-0.89) | 0.39 (0.05-3.18) |  |
| Most/all of the time | 20 (12.7) | 5 (17.2) | 1.10 (0.69-1.75) | 1.70 (0.56-5.15) |  | 1.24 (0.75-2.05) | 1.96 (0.57-6.79) | *0.43* |

Note: Among those who are black and white only (n=32,046). Due to small sample size, “Other” race (n=1215) was excluded. Analysis is complete case only (thus observations with missing covariates or exposure of interest were excluded).

HR: hazard ratio; 95% CI: 95% confidence interval

^a^ HR was adjusted for age at enrollment

^b^ HR was additionally adjusted for enrollment source, education, income, employment status, BMI, depression, alcohol drinking, smoking status, diabetes, hypertension, COPD, asthma, stroke, total physical activity, and BPH.

^c^ The death numbers reflect the fully adjusted model numbers.

## **Table S7. Multiple Imputation: Associations between sleep characteristics at enrollment and overall prostate cancer-specific mortality in the SCCS**

|  | Age and race/ethnicity adjusted^a^ | Fully adjusted^b^ |
| --- | --- | --- |
|  | Overall | Overall |
|  | HR (95% CI) | HR (95% CI) |
| Sleep duration, hr |  |  |
| Sleep average |  |  |
| <6 | 0.95 (0.62-1.48) | 0.95 (0.61-1.48) |
| 6 | 0.90 (0.62-1.31) | 0.89 (0.61-1.30) |
| 7-8 | 1.00 (Ref) | 1.00 (Ref) |
| ≥9 | 1.32 (0.94-1.84) | 1.31 (0.93-1.85) |
|  | *p trend=0.12* | *p trend=0.12* |
|  |  |  |
| Weekday |  |  |
| <6 | 0.79 (0.51-1.23) | 0.79 (0.51-1.24) |
| 6 | 0.85 (0.60-1.21) | 0.86 (0.60-1.22) |
| 7-8 | 1.00 (Ref) | 1.00 (Ref) |
| ≥9 | 1.27 (0.89-1.83) | 1.27 (0.88-1.84) |
|  | *p trend=0.04* | *p trend=0.04* |
|  |  |  |
| Weekend |  |  |
| <6 | 1.10 (0.70-1.71) | 1.07 (0.68-1.68) |
| 6 | 1.23 (0.86-1.77) | 1.20 (0.83-1.73) |
| 7-8 | 1.00 (Ref) | 1.00 (Ref) |
| ≥9 | 1.45 (1.04-2.01) | 1.45 (1.05-2.02) |
|  | *p trend= 0.36* | *p trend= 0.28* |
|  |  |  |
| Restless sleep |  |  |
| Rarely/none of the time | 1.00 (Ref) | 1.00 (Ref) |
| Some of the time | 0.88 (0.66-1.18) | 0.89 (0.66-1.20) |
| Much of the time | 0.54 (0.29-1.01) | 0.56 (0.30-1.05) |
| Most/all of the time | 1.23 (0.80-1.88) | 1.23 (0.80-1.91) |

HR: hazard ratio; 95% CI: 95% confidence interval

^a^ HR was adjusted for age at enrollment and race/ethnicity

^b^ HR was additionally adjusted for enrollment source, education, income, employment status,

BMI, depression, alcohol drinking, smoking status, diabetes, hypertension, COPD, asthma,

stroke, total physical activity, and BPH.

## **Table S8. Multiple Imputation: Associations between sleep characteristics at enrollment and overall prostate cancer-specific mortality by race in the SCCS**

|  | Age-adjusted^a^ | |  | Race/ethnicity-specific (fully adjusted)^b^ | | |
| --- | --- | --- | --- | --- | --- | --- |
|  | **Non-Hispanic Black** | **Non-Hispanic White** |  | **Non-Hispanic Black** | **Non-Hispanic White** | **p interaction** |
|  | HR (95% CI) | HR (95% CI) |  | HR (95% CI) | HR (95% CI) |  |
| Sleep duration, hr |  |  |  |  |  |  |
| Sleep average |  |  |  |  |  |  |
| <6 | 0.92 (0.57-1.49) | 1.91 (0.64-5.65) |  | 0.94 (0.58-1.54) | 1.41 (0.45-4.36) |  |
| 6 | 0.88 (0.58-1.35) | 1.04 (0.41-2.60) |  | 0.89 (0.58-1.37) | 0.99 (0.39-2.52) |  |
| 7-8 | 1.00 (Ref) | 1.00 (Ref) |  | 1.00 (Ref) | 1.00 (Ref) |  |
| ≥9 | 1.30 (0.90-1.89) | 1.34 (0.50-3.60) |  | 1.27 (0.87-1.85) | 1.29 (0.47-3.54) | *0.62* |
|  | *p trend=0.13* | *p trend=0.63* |  | *p trend=0.19* | *p trend=0.91* |  |
|  |  |  |  |  |  |  |
| Weekday |  |  |  |  |  |  |
| <6 | 0.75 (0.46-1.23) | 1.77 (0.59-5.29) |  | 0.77 (0.47-1.27) | 1.39 (0.45-4.30) |  |
| 6 | 0.81 (0.55-1.21) | 1.27 (0.55-2.93) |  | 0.83 (0.56-1.24) | 1.27 (0.54-2.95) |  |
| 7-8 | 1.00 (Ref) | 1.00 (Ref) |  | 1.00 (Ref) | 1.00 (Ref) |  |
| ≥9 | 1.29 (0.87-1.91) | 1.29 (0.44-3.83) |  | 1.25 (0.84-1.86) | 1.28 (0.42-3.86) |  |
|  | *p trend=0.03* | *p trend=0.47* |  | *p trend=0.06* | *p trend=0.68* | *0.39* |
|  |  |  |  |  |  |  |
| Weekend |  |  |  |  |  |  |
| <6 | 1.04 (0.63-1.70) | 1.90 (0.55-6.57) |  | 1.04 (0.63-1.72) | 1.35 (0.37-4.93) |  |
| 6 | 1.15 (0.76-1.75) | 1.80 (0.77-4.22) |  | 1.15 (0.76-1.75) | 1.68 (0.70-3.99) |  |
| 7-8 | 1.00 (Ref) | 1.00 (Ref) |  | 1.00 (Ref) | 1.00 (Ref) |  |
| ≥9 | 1.43 (1.00-2.06) | 1.65 (0.68-4.01) |  | 1.43 (0.99-2.05) | 1.68 (0.68-4.14) | *0.66* |
|  | *p trend=0.26* | *p trend=0.54* |  | *p trend=0.27* | *p trend=0.92* |  |
|  |  |  |  |  |  |  |
| Restless sleep |  |  |  |  |  |  |
| Rarely/none of the time | 1.00 (Ref) | 1.00 (Ref) |  | 1.00 (Ref) | 1.00 (Ref) |  |
| Some of the time | 0.82 (0.59-1.14) | 1.50 (0.70-3.20) |  | 0.82 (0.59-1.14) | 1.57 (0.73-3.39) |  |
| Much of the time | 0.37 (0.16-0.85) | 0.37 (0.05-2.89) |  | 0.39 (0.17-0.91) | 0.34 (0.04-2.69) |  |
| Most/all of the time | 1.21 (0.76-1.94) | 2.07 (0.71-6.02) |  | 1.24 (0.77-2.01) | 1.78 (0.58-5.46) | *0.54* |

Note: Among those who are black and white only. Due to small sample size, “Other” race/ethnicity was excluded.

HR: hazard ratio; 95% CI: 95% confidence interval

a HR was adjusted for age at enrollment

b HR was additionally adjusted for enrollment source, education, income, employment status, BMI, depression, alcohol drinking, smoking status, diabetes, hypertension, COPD, asthma, stroke, total physical activity, and BPH.

## **Table S9. Sensitivity Analyses: Associations between sleep characteristics at enrollment and prostate cancer-specific mortality in the SCCS, additionally adjusted for PSA screening, DRE screening, HEI-2010, and family history of prostate cancer**

|  | Cohort  N=33,568 | Deaths  N=225 | Age and race/ethnicity adjusted^a^ | Fully adjusted^b^ |
| --- | --- | --- | --- | --- |
|  | Overall | Overall | Overall | Overall |
|  | N (%)^c^ | N (%)^c^ | HR (95% CI) | HR (95% CI) |
| Sleep duration, hr |  |  |  |  |
| Sleep average |  |  |  |  |
| <6 | 3592 (14.0) | 17 (10.4) | 0.91 (0.58-1.42) | 0.93 (0.55-1.58) |
| 6 | 5219 (20.3) | 31 (18.9) | 0.90 (0.62-1.30) | 1.02 (0.67-1.55) |
| 7-8 | 12393 (48.2) | 77 (47.0) | 1.00 (Ref) | 1.00 (Ref) |
| ≥9 | 4520 (17.6) | 39 (23.8) | 1.31 (0.93-1.83) | 1.44 (0.97-2.13) |
|  |  |  | *p trend=0.10* | *p trend=0.14* |
|  |  |  |  |  |
| Weekday |  |  |  |  |
| <6 | 3957 (15.4) | 16 (9.8) | 0.76 (0.48-1.19) | 0.76 (0.43-1.27) |
| 6 | 6331 (24.6) | 37 (22.6) | 0.85 (0.60-1.21) | 1.00 (0.67-1.47) |
| 7-8 | 12135 (47.1) | 81 (49.4) | 1.00 (Ref) | 1.00 (Ref) |
| ≥9 | 3355 (13.0) | 30 (18.3) | 1.26 (0.88-1.82) | 1.31 (0.86-2.01) |
|  |  |  | *p trend=0.03* | *p trend=0.11* |
|  |  |  |  |  |
| Weekend |  |  |  |  |
| <6 | 3580 (13.9) | 14 (8.5) | 1.05 (0.67-1.65) | 0.81 (0.45-1.46) |
| 6 | 4964 (19.3) | 37 (22.6) | 1.23 (0.85-1.77) | 1.39 (0.92-2.08) |
| 7-8 | 11512 (44.7) | 66 (40.2) | 1.00 (Ref) | 1.00 (Ref) |
| ≥9 | 5721 (22.2) | 47 (28.7) | 1.44 (1.04-2.00) | 1.55 (1.06-2.26) |
|  |  |  | *p trend= 0.29* | *p trend= 0.13* |
|  |  |  |  |  |
| Restless sleep |  |  |  |  |
| Rarely/none of the time | 9497 (36.8) | 75 (46.0) | 1.00 (Ref) | 1.00 (Ref) |
| Some of the time | 10219 (39.6) | 61 (37.4) | 0.88 (0.66-1.18) | 0.89 (0.63-1.26) |
| Much of the time | 2905 (11.3) | 9 (5.5) | 0.55 (0.29-1.02) | 0.63 (0.31-1.27) |
| Most/all of the time | 3184 (12.3) | 18 (11.0) | 1.18 (0.77-1.82) | 1.09 (0.64-1.85) |

Note: Of the 33,568 participants in the analytic cohort, 7,677 were excluded from the fully adjusted models for each exposure due to any missing covariate data.

HR: hazard ratio; 95% CI: 95% confidence interval

^a^ HR was adjusted for age at enrollment and race/ethnicity

^b^ HR was additionally adjusted for enrollment source, education, income, employment status, BMI, depression, alcohol drinking, smoking status, diabetes, hypertension, COPD, asthma, stroke, total physical activity, BPH, HEI-2010, family history (father and brother) of prostate cancer, PSA screening, and DRE screening.

^c^ The cohort and death numbers reflect the fully adjusted model numbers.

## **Table S10. Sensitivity Analyses: Associations between sleep characteristics at enrollment and prostate cancer-specific mortality by race/ethnicity in the SCCS, additionally adjusted for PSA screening, DRE screening, HEI-2010, and family history of prostate cancer (cohort N=32,046)**

|  | Deaths (Black) N=178 | Deaths (White) N=34 | Age-adjusted^a^ | |  | Race/ethnicity-specific (fully adjusted)^b^ | | | |
| --- | --- | --- | --- | --- | --- | --- | --- | --- | --- |
|  |  |  | Non-Hispanic Black | Non-Hispanic White |  | Non-Hispanic Black | Non-Hispanic White | p interaction |  |
|  | N (%)^c^ | N (%)^c^ | HR (95% CI) | HR (95% CI) |  | HR (95% CI) | HR (95% CI) |  |  |
| Sleep duration, hr |  |  |  |  |  |  |  |  |  |
| Sleep average |  |  |  |  |  |  |  |  |  |
| <6 | 15 (11.7) | 2 (7.4) | 0.88 (0.54-1.44) | 1.86 (0.63-5.54) |  | 1.00 (0.56-1.78) | 0.81 (0.17-3.77) |  |  |
| 6 | 23 (18.0) | 6 (22.2) | 0.88 (0.58-1.35) | 1.04 (0.41-2.61) |  | 1.00 (0.61-1.62) | 1.28 (0.48-3.41) |  |  |
| 7-8 | 57 (44.5) | 14 (51.9) | 1.00 (Ref) | 1.00 (Ref) |  | 1.00 (Ref) | 1.00 (Ref) |  |  |
| ≥9 | 33 (25.8) | 5 (18.5) | 1.29 (0.89-1.87) | 1.34 (0.50-3.59) |  | 1.40 (0.91-2.16) | 1.87 (0.65-5.40) | *0.75* |  |
|  |  |  | *p trend=0.10* | *p trend=0.66* |  | *p trend=0.26* | *p trend=0.49* |  |  |
|  |  |  |  |  |  |  |  |  |  |
| Weekday |  |  |  |  |  |  |  |  |  |
| <6 | 14 (10.9) | 2 (7.4) | 0.72 (0.44-1.19) | 1.74 (0.58-5.20) |  | 0.82 (0.46-1.47) | 0.76 (0.16-3.51) |  |  |
| 6 | 28 (21.9) | 7 (25.9) | 0.81 (0.55-1.21) | 1.27 (0.55-2.93) |  | 0.99 (0.63-1.56) | 1.41 (0.56-3.57) |  |  |
| 7-8 | 60 (46.9) | 14 (51.9) | 1.00 (Ref) | 1.00 (Ref) |  | 1.00 (Ref) | 1.00 (Ref) |  |  |
| ≥9 | 26 (20.3) | 4 (14.8) | 1.28 (0.87-1.90) | 1.29 (0.44-3.82) |  | 1.31 (0.83-2.09) | 1.87 (0.58-5.95) | *0.85* |  |
|  |  |  | *p trend=0.02* | *p trend=0.49* |  | *p trend=0.19* | *p trend=0.60* |  |  |
|  |  |  |  |  |  |  |  |  |  |
|  |  |  |  |  |  |  |  |  |  |
|  | N (%)^c^ | N (%)^c^ | HR (95% CI) | HR (95% CI) |  | HR (95% CI) | HR (95% CI) |  |  |
| Weekend |  |  |  |  |  |  |  |  |  |
| <6 | 11 (8.6) | 2 (7.4) | 0.99 (0.60-1.64) | 1.84 (0.53-6.39) |  | 0.75 (0.39-1.45) | 1.08 (0.22-5.20) |  |  |
| 6 | 28 (21.9) | 7 (25.9) | 1.15 (0.76-1.74) | 1.80 (0.77-4.21) |  | 1.31 (0.82-2.09) | 2.06 (0.78-5.45) |  |  |
| 7-8 | 50 (39.1) | 11 (40.7) | 1.00 (Ref) | 1.00 (Ref) |  | 1.00 (Ref) | 1.00 (Ref) |  |  |
| ≥9 | 39 (30.5) | 7 (25.9) | 1.42 (0.99-2.04) | 1.65 (0.68-4.01) |  | 1.46 (0.96-2.22) | 2.68 (1.01-7.08) | *0.90* |  |
|  |  |  | *p trend=0.21* | *p trend=0.57* |  | *p trend=0.14* | *p trend=0.54* |  |  |
|  |  |  |  |  |  |  |  |  |  |
| Restless sleep |  |  |  |  |  |  |  |  |  |
| Rarely/none of the time | 63 (49.6) | 9 (33.3) | 1.00 (Ref) | 1.00 (Ref) |  | 1.00 (Ref) | 1.00 (Ref) |  |  |
| Some of the time | 46 (36.2) | 13 (48.2) | 0.82 (0.59-1.14) | 1.50 (0.70-3.19) |  | 0.80 (0.54-1.17) | 1.47 (0.62-3.50) |  |  |
| Much of the time | 4 (3.2) | 1 (3.7) | 0.37 (0.16-0.86) | 0.37 (0.05-2.89) |  | 0.35 (0.13-0.97) | 0.39 (0.05-3.25) |  |  |
| Most/all of the time | 14 (11.0) | 4 (14.8) | 1.17 (0.73-1.88) | 2.01 (0.69-5.88) |  | 1.05 (0.58-1.90) | 1.64 (0.46-5.83) | *0.44* |  |

Note: Among those who are black and white only (n=32,046). Due to small sample size, “Other” race (n=1215) was excluded. Analysis is complete case only (thus observations with missing covariates or exposure of interest were excluded). Of the 22,437 black participants and 9,609 white participants in the analytic cohort, 5,089 were excluded from the fully adjusted models for each exposure due to any missing covariate data for black men, and 1,996 were excluded from the fully adjusted models for each exposure due to any missing covariate data for white men.

Multiple comparisons were adjusted using the Bonferroni method, and results were not significant

HR: hazard ratio; 95% CI: 95% confidence interval

^a^ HR was adjusted for age at enrollment

^b^ HR was additionally adjusted for enrollment source, education, income, employment status, BMI, depression, alcohol drinking, smoking status, diabetes, hypertension, COPD, asthma, stroke, total physical activity, BPH, HEI-2010, family history (father and brother) of prostate cancer, PSA screening, and DRE screening.

^c^ The death numbers reflect the fully adjusted model numbers.

## **References**

1. Anukam DC, Nianogo RA, Arah OA, et al. Sleep Duration and Prostate Cancer Risk in the Southern Community Cohort Study. *Cancer Med*. 2026;15(1):e71466. doi:10.1002/cam4.71466
